# The epicardial adipose tissue confined in the atrioventricular groove can be used to assess atrial adipose tissue and atrial dysfunction in cardiac magnetic resonance imaging

**DOI:** 10.1093/ehjimp/qyae057

**Published:** 2024-06-14

**Authors:** Jonathan Bialobroda, Khaoula Bouazizi, Maharajah Ponnaiah, Nadjia Kachenoura, Etienne Charpentier, Mohamed Zarai, Karine Clement, Fabrizio Andreelli, Judith Aron-Wisnewsky, Stéphane N Hatem, Alban Redheuil

**Affiliations:** Institute of Cardiology, Foundation for Innovation in Cardiometabolism and Nutrition—ICAN, INSERM UMRS 1166, Sorbonne Université, AP-HP Pitié-Salpêtrière University Hospital, 47-83, Boulevard de l’Hôpital, 75013 Paris, France; Laboratoire d’Imagerie Biomédicale, CNRS, INSERM UMR 1146, Sorbonne Université, Paris, France; Foundation for Innovation in Cardiometabolism and Nutrition (IHU-ICAN, ANR-10-IAHU-05), 47-83, Boulevard de l’Hôpital, 75013 Paris, France; Foundation for Innovation in Cardiometabolism and Nutrition (IHU-ICAN, ANR-10-IAHU-05), 47-83, Boulevard de l’Hôpital, 75013 Paris, France; Laboratoire d’Imagerie Biomédicale, CNRS, INSERM UMR 1146, Sorbonne Université, Paris, France; Institute of Cardiometabolism and Nutrition—ICAN, INSERM UMR 1146, Laboratoire d’Imagerie Biomédicale, Unité D’Imagerie Cardiovasculaire et Thoracique, Sorbonne Université, AP-HP Hôpital Pitié-Salpêtrière, Paris, France; Institute of Cardiometabolism and Nutrition—ICAN, INSERM UMR 1146, Laboratoire d’Imagerie Biomédicale, Unité D’Imagerie Cardiovasculaire et Thoracique, Sorbonne Université, AP-HP Hôpital Pitié-Salpêtrière, Paris, France; Nutrition and Obesity: Systemic Approaches, NutriOmics, Sorbonne Université, INSERM, Paris, France; Department of Nutrition, Sorbonne Université, Assistance Publique- Hôpitaux de Paris, AP-HP, Pitié-Salpêtrière University Hospital, 47-83 Boulevard de l'Hôpital, 75013 Paris, France; Nutrition and Obesity: Systemic Approaches, NutriOmics, Sorbonne Université, INSERM, Paris, France; Department of Diabetology, AP-HP Pitié-Salpêtrière University Hospital, Sorbonne Université, Paris, France; Nutrition and Obesity: Systemic Approaches, NutriOmics, Sorbonne Université, INSERM, Paris, France; Department of Nutrition, Sorbonne Université, Assistance Publique- Hôpitaux de Paris, AP-HP, Pitié-Salpêtrière University Hospital, 47-83 Boulevard de l'Hôpital, 75013 Paris, France; Institute of Cardiology, Foundation for Innovation in Cardiometabolism and Nutrition—ICAN, INSERM UMRS 1166, Sorbonne Université, AP-HP Pitié-Salpêtrière University Hospital, 47-83, Boulevard de l’Hôpital, 75013 Paris, France; Foundation for Innovation in Cardiometabolism and Nutrition (IHU-ICAN, ANR-10-IAHU-05), 47-83, Boulevard de l’Hôpital, 75013 Paris, France; Institute of Cardiometabolism and Nutrition—ICAN, INSERM UMR 1146, Laboratoire d’Imagerie Biomédicale, Unité D’Imagerie Cardiovasculaire et Thoracique, Sorbonne Université, AP-HP Hôpital Pitié-Salpêtrière, Paris, France

**Keywords:** epicardial adipose tissue, magnetic resonance imaging CMR, atrial cardiomyopathy, cardiometabolism, left atrial strain

## Abstract

**Aims:**

The growing interest in epicardial adipose tissue (EAT) as a biomarker of atrial fibrillation is limited by the difficulties in isolating EAT from other paracardial adipose tissues. We tested the feasibility and value of measuring the pure EAT contained in the atrioventricular groove (GEAT) using cardiovascular magnetic resonance (CMR) imaging in patients with distinct metabolic disorders.

**Methods and results:**

CMR was performed on 100 patients from the MetaCardis cohort: obese (*n* = 18), metabolic syndrome (MSD) (*n* = 25), type-2 diabetes (T2D) (*n* = 42), and age- and gender-matched healthy controls (*n* = 15). GEAT volume measured from long-axis views was obtained in all patients with a strong correlation between GEAT and atrial EAT (*r* = 0.95; *P* < 0.0001). GEAT volume was higher in the three groups of patients with metabolic disorders and highest in the MSD group compared with controls. GEAT volume, as well as body mass and body fat, allowed obese, T2D, and MSD patients to be distinguished from controls. GEAT T1 relaxation and peak longitudinal left atrial (LA) strain in CMR were decreased in T2D patients. Logistic regression and random forest machine learning methods were used to create an algorithm combining GEAT volume, GEAT T1, and peak LA strain to identify T2D patients from other groups with an area under curve (AUC) of 0.81 (Se: 77%, Spe: 80%; 95% confidence interval 0.72–0.91, *P* < 0.0001).

**Conclusion:**

Atrioventricular groove adipose tissue characteristics measured during routine CMR can be used as a proxy of atrial EAT and integrated in a multi-parametric CMR biomarker for early identification of atrial cardiomyopathy.

## Structured Graphical Abstract

**Structured Graphical Abstract:**
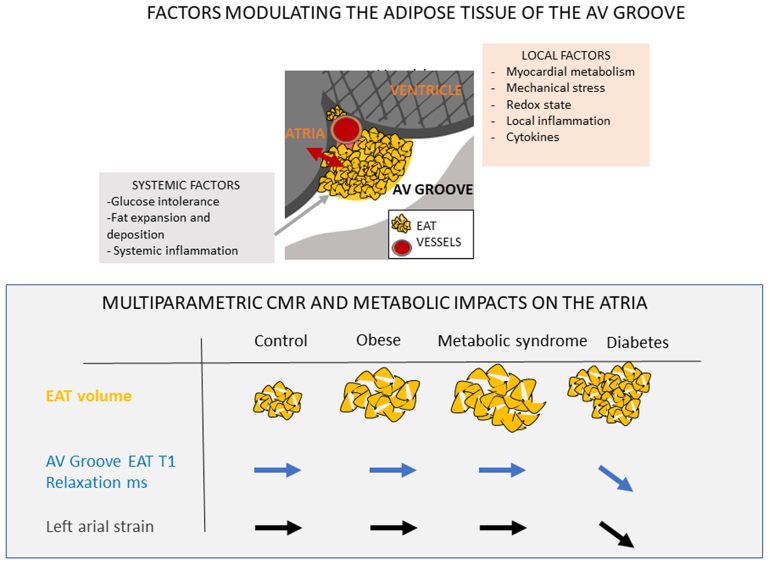

## Introduction

Epicardial adipose tissue (EAT) has emerged as an important pathogenic factor for a host of cardiac diseases including coronary artery disease, heart failure—notably with preserved ejection fraction—and atrial fibrillation (AF).^1^ In particular, the EAT volume has been shown to be associated with the increase in both incidence and rate of recurrence of AF.^2,3^

EAT, the external component of the epicardium, is distinct from pericardial fat located outside the parietal face of the pericardium by both its embryologic origin and biological properties. This ectopic visceral ‘brown’ adipose tissue is a major source of free fatty acids and a myriad of adipokines that can freely diffuse in the neighbouring myocardium regulating its metabolic and oxidative phenotypes.^1^ However, in specific clinical circumstances, EAT can produce pro-atherogenic or inflammatory adipokines^4,5^ contributing to tissue remodelling.^5^ In addition, EAT in the subepicardial layers of the atrial myocardial wall can become fibrotic contributing to the substrate of AF.^6^

Taking these factors into account, there is a general interest in the possibility of considering EAT in clinical practice as a candidate biomarker of early onset and progression of cardiac diseases, including AF, as demonstrated with the pericoronary fat attenuation index and the severity of coronary artery atherosclerosis.^7^ Consequently, current research efforts are dedicated to the development of imaging techniques to accurately measure EAT using ultrasounds, computed tomography (CT), and cardiac magnetic resonance (CMR) imaging.^8^ CT and CMR have the advantage over ultrasounds as they are able to do a 3D assessment of cardiac adipose tissues and characterize EAT content in terms of the nature of lipid concentration and/or the composition of fat components. Nevertheless, the precise delineation between epicardial, pericardial, and extrapericardial adipose tissues remains challenging and time-consuming, as this requires expert reading and manual intervention. Whereas *ex vivo* CMR has been shown to allow the differentiation and quantification of fat, fibrosis, and fibro-fatty components, clinical application remains hampered by insufficient spatial resolution *in vivo.*^9^ The lack of standardized and quantitatively validated imaging approaches to quantify EAT, which is scattered at the surface of the four chambers of the heart, remains a drawback.

A distinct adipose tissue is constantly present in the atrioventricular (AV) groove (GEAT). This adipose tissue is confined in a well-delineated anatomic area of the heart and is defined by proximity with both atrial and ventricular myocardium and the left circumflex and right coronary arteries and veins, thus easier to quantify using imaging. It has been clearly established using lineage tracing mouse models that the adipose tissue of the AV groove originates from the epicardium where quiescent epicardial progenitor cells are niched in adult hearts.^10,11^

The present study was undertaken to first determine whether the EAT of the AV groove (GEAT) can be measured using a standardized clinical CMR protocol and if it can be used as a surrogate of atrial EAT volume. In addition, we tested a multi-parametric CMR score including GEAT characteristics in a cohort of patients with distinct metabolic disorders to detect subclinical atrial alterations.

## Methods

### Patient cohort

One hundred patients from the MetaCardis (Metagenomics and Integrative Systems Medicine of Cardiometabolic Diseases) cohort, sponsored by the 7th Framework Program of the European Commission (https://cordis.europa.eu/project/id/305312), were included from June 2013 to June 2016 in an ancillary CMR study. Participants were divided into four groups based on distinct metabolic profiles: metabolic syndrome (MSD) (defined by the International Diabetes Federation criteria, *n* = 25), severe obesity (body mass index ≥ 35 kg/m^2^, *n* = 18), type-2 diabetes (T2D, *n* = 42), and age- and gender-matched healthy controls (*n* = 15). The patients did not have overt cardiovascular disease and had no previous history of cardiac structural diseases; they were all in sinus rhythm with normal cardiac function and morphology, notably no left atrial (LA) dilation (LA volume < 34 mL/m2) in echocardiography. The control group was composed of asymptomatic individuals without clinical and biological evidence of metabolic disorders or weight issues.

All participants had CMR after inclusion. All participants were free of overt cardiovascular disease and remained in sinus rhythm at the time of CMR. All participants provided informed consent, and the study protocol was approved by the local Internal Review Board. Inclusion criteria are provided online via the following link: https://clinicaltrials.gov/ct2/show/NCT02059538.

### Cardiovascular magnetic resonance

**Figure 1:**
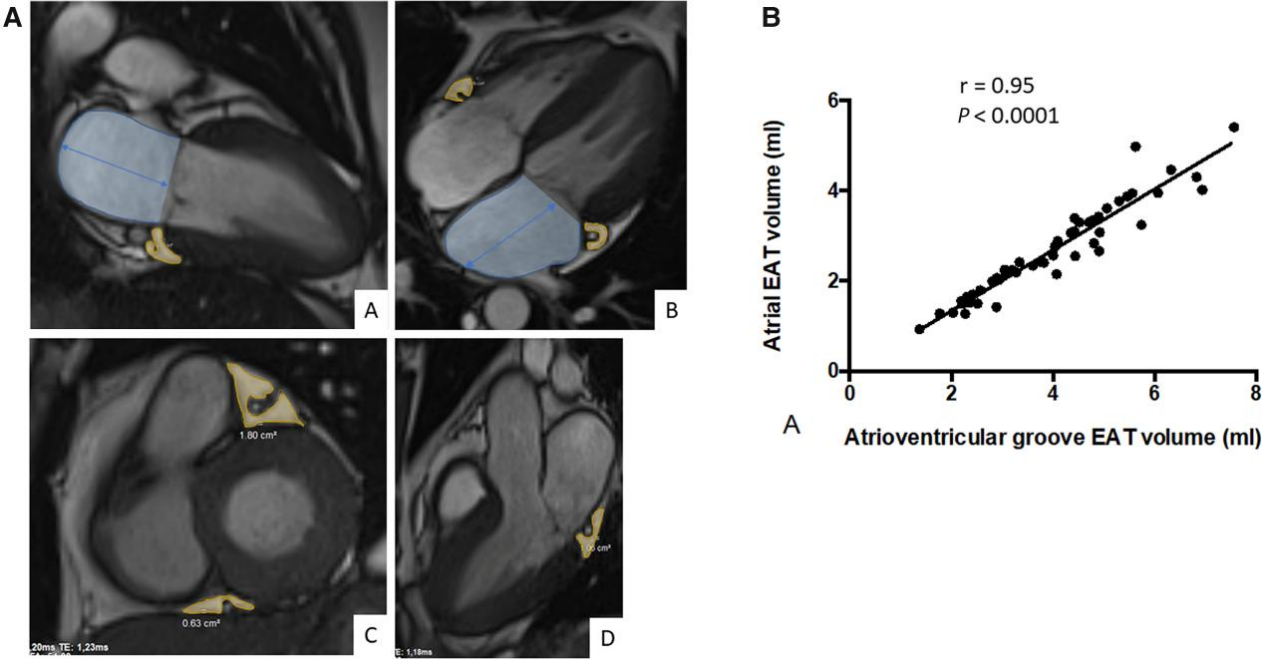
Left atrial CMR measurements (*A*). Long-axis SSFP cine views (two-chamber, three-chamber, and four-chamber) and basal short-axis view showing GEAT measurement in the AV groove (yellow surface) and left atrial surface and length measurements (blue surface overlay and bidirectional arrow). Relationship of total atrial EAT to GEAT volumes (*B*).

All subjects underwent a dedicated CMR protocol performed with a clinical 1.5 T magnet (Aera; Siemens Healthineers, Erlangen, Germany). Cardiac function was assessed using pre-contrast cine steady-state free-precession (SSFP) sequences in two, three, and four-chamber long-axis orientations and complete short-axis stack with 10–12 acquisitions, one per breath hold, to maximize imaging quality, covering the entire left ventricle (LV) from the mitral annulus to the apex. Typical acquisition parameters were slice thickness 8 mm, matrix: 286 × 286 in-plane, repetition time: 2.6 ms, echo time: 1.2 ms, flip angle = 60°, and number of cardiac phases = 25/cycle. T1 relaxation times were assessed using the Modified Look-Locker Imaging (MOLLI) sequence with a 5(3)3 scheme acquired before and 15 min after the injection of 0.02 mmol/kg of gadobenate dimeglumine (MultiHance). Note that as cardiac MRI exams were performed prior to 2017 and until this date, MultiHance was used as standard of care in our department. The QMass software (Medis Suite, the Netherlands) was used for the measurement of ventricular volumes and LV mass as well as LA volumes. LA end-diastolic volume was estimated using the established biplanar area-length method (*Figure 1A*).

### Atrial and AV groove EAT volumes

Total and groove adipose tissue volumes and myocardial and groove adipose tissue T1 relaxation times were measured using the QMass software (*Figure 1A* and *3A*). AV groove EAT volume was estimated by manual delineation from end-diastolic cine SSFP images in the standardized recommended long-axis views: four-chamber (right and left lateral AV grooves), three-chamber (left lateral AV groove), two-chamber (inferior AV groove), and basal short-axis view (anterior and posterior AV groove). The basal short-axis view was defined as recommended as the plane between the tip of the mitral valve and the tip of the papillary muscles. The AV groove adipose tissue having a triangular or nodular shape and appearing hyperintense on SSFP images delineated by a dark line corresponding to chemical shift at the outer fat/pericardial fluid interface was segmented manually on long-axis images excluding vessels (*Figure 1A*). Atrial EAT volume was measured from four-chamber (lateral LA and inter-atrial septum), three-chamber (lateral), and two-chamber views (inferior and superior LA) in 55 patients to be compared with GEAT. All EAT values were expressed in millilitres, after multiplying the measured surface areas by the acquisition slice thickness.

### Native T1 mapping

Native T1 of GEAT was measured by manual delineation in the anterior AV groove on the basal short-axis T1 parametric map excluding the coronary vessels in 94 patients of the cohort (six patients did not have the adequate sequences; *Figure 3A*). T1 relaxation times are expressed in milliseconds.

### LA feature tracking

LA feature tracking was performed by using a dedicated cardiac MR imaging feature-tracking software,^12^ from two- and four-chamber cine CMR images (*Figure 3B*). LA myocardial deformation was assessed as peak global longitudinal strains (GLS), as recommended by the European Association of Cardiovascular Imaging and the American Society of Echocardiography.^13^ GLS was calculated by averaging the strain values obtained in the long-axis two- and four-chamber views. Longitudinal strain was defined as the temporal variation of the LA contour length. The endocardial borders, excluding pulmonary veins and LA appendage, were manually traced for initialization. The inter-observer variability analysis for this method was previously reported with coefficients of variation of 4.3%.^14^ The analysed metrics were peak longitudinal atrial strain, corresponding to the maximal atrial reservoir deformation (starts at the end of ventricular diastole with mitral valve closure and continues until mitral valve opening), and pre-A-wave longitudinal strain corresponding to the LA booster pump function (occurs from the onset of LA contraction until the end of ventricular diastole in patients with sinus rhythm). Strain values are expressed in percent.

### Statistical analysis

Data are shown either as mean ± SEM or median [interquartile range (IQR)] for continuous variables and as number (percentage) for categorical variables. The Shapiro–Wilk test was used to test normal distribution of data. For comparisons of means between groups, one-way analysis of variance (ANOVA) tests (with Bonferroni correction) and Kruskal–Wallis tests were performed. Error bars on all figures represent standard deviations from the mean values.

Intra-observer variability of measurement was evaluated by analysis of the intra-class correlation that was equal to 0.97 [95% confidence interval (CI) 0.95–0.98, *P* < 0.0001] for GEAT volume and 0.90 (95% CI 0.95–0.98, *P* < 0.0001) for GEAT T1. Inter-observer variability for GEAT volume was evaluated by analysis of the intra-class correlation coefficient that was equal to 0.98 (95% CI 0.96–0.99, *P* < 0.0001). The two measurements were performed by two different readers blinded to all study results or patient characteristics with an identical method, and time interval between the two measurements was 1 year.

Linear regression analysis was used to assess the impact of metabolic disorder groups on LA strain, adjusting for myocardial mass, age, LA volumes, and sex. To ensure the independence of predictors, multi-collinearity was assessed using variance inflation factors (VIFs), with a VIF value greater than 3 indicating potential issues requiring further investigation. Model assumptions were verified through diagnostic checks including analysis of residuals for homoscedasticity and normality. Statistical significance was set at *P* < 0.05. Analyses were performed using SPSS software, version 28.0.

Pearson correlation coefficients from linear regression between continuous variables were provided. Multivariable analysis was performed using logistic regression by the backward conditional method. The variables used in the multivariable analysis were chosen based on their univariate significance (*P* < 0.05). The multivariable analysis was validated using the Omnibus Test of Model Coefficients and receiver operating characteristic (ROC) analysis. Prediction scores were developed for each model from the coefficient values of significant variables that were retained in the backward conditional method, along with the coefficient of the constant. ROC curves were plotted to check for the sensitivity and specificity of the scores to predict the outcomes. Random forest machine learning algorithm was applied to validate the models and predict the power of the prognostic score. The statistical significance was set to *P* < 0.05 for all the tests. All statistical analysis was performed using PRISM 6 (GraphPad Software Inc., Canada), SPSS software, version 28.0 (IBM Corp., Armonk, NY, USA), and R software version 4.1.3.

## Results

### Adipose tissue of the AV groove can be analysed from routine CMR

Measurement of the adipose tissue located in the AV groove, GEAT, using CMR was feasible in all patients using standard long- and short-axis cine acquisition views. Intra-observer variability of measurement was evaluated by analysis of the intra-class correlation, which was equal to 0.97 (95% CI 0.95–0.98, *P* < 0.0001) for GEAT volume and 0.90 (95% CI 0.95–0.98, *P* < 0.0001) for GEAT T1. There was a high linear correlation between GEAT and atrial EAT volumes as indicated by a correlation coefficient of 0.95 (*P* < 0.0001; *Figure 1B*). Taken together, these results indicate that adipose tissue of the AV groove can be analysed and quantified in CMR and that GEAT can used as a proxy of EAT.

### The AV groove adipose tissue parameters vary according to the metabolic status of patients

**Figure 2:**
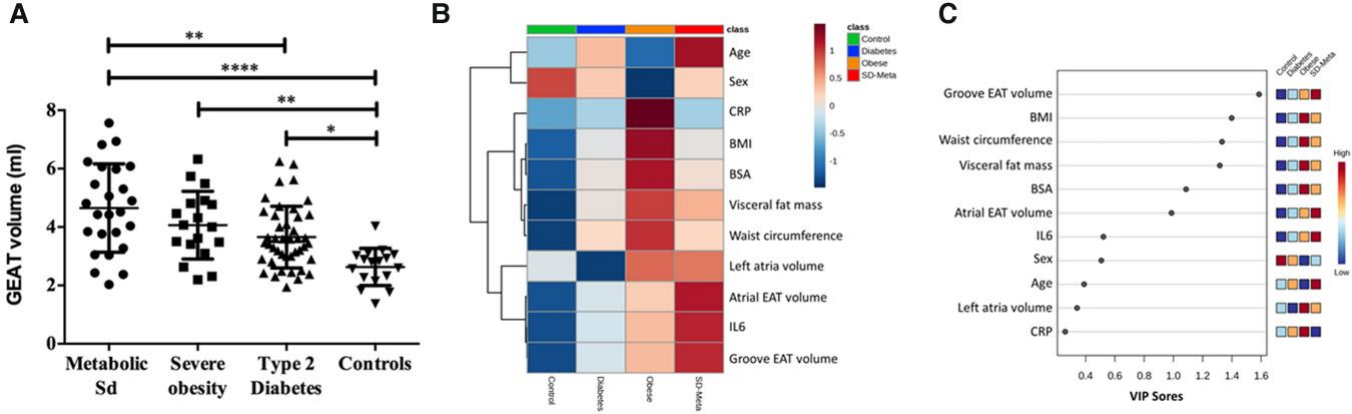
Distribution of GEAT volumes by cardiometabolic group (*A*); heatmaps showing correlations between demographic, metabolic, and inflammation factors and GEAT volume (*B*); VIP scores for explanatory variables according to cardiometabolic groups (*C*).

GEAT volume was described in different patient groups according to the severity of metabolic alterations and obesity: patients with severe obesity, with MSD or with T2D as compared with healthy controls. Patient characteristics are summarized in *Table 1*. The volume of GEAT displayed an important scatter of values. GEAT volume was higher in the three groups of patients suffering from a metabolic disorder compared with the control group (*Figure 2A*). Furthermore, patients with MSD had the highest volume of GEAT [4.5 mL, (IQR 3.5–6.0 mL)] compared with controls, but this trend did not reach statistical significance when compared with patients with obesity [4.0 mL, (IQR 3.3–4.8 mL)] and T2D [3.5 mL, (IQR 3.0–4.4 mL)]. The correlations between GEAT and other parameters of anthropometry, body composition and distribution of fat tissue, inflammation, and LA characteristics were studied using heat maps. There was a positive correlation between increased GEAT volume and body mass index (0.30), visceral fat (0.37), waist circumference (0.30), age (0.19) and inflammation as seen with interleukin (IL)-6 (0.21). Note that results were adjusted for sex. Then, using these parameters, it was possible to identify obese, T2D, MSD, and control patients *a posteriori* by performing a partial least squares-discriminant analysis (PLS-DA) (*Figure 2B*). Moreover, the volume of GEAT was the primary contributor for defining the patient group, as indicated by the variable importance in the projection score analysis (*Figure 2C*).

**Table 1:** Patient characteristics and summary of biological and CMR parameters according to cardiometabolic group

	Controls	Patients with metabolic syndrome	Patients with obesity	Patients with diabetes	(*P*)*	Pairwise comparisons for diabetes*
*n*	15	25	18	42		
**Demographics and morphometry**						
Age (years)	54 (46–63)	61 (52–66)	49 (41–59)	57 (52–62)	0.100	
Sex F/M (*n*)	4/11	11/14	15/3	19/23	0.006	
Weight (kg)	68 (65–72)	86 (78–102)	110 (100–119)	88 (77–99)	**<0**.**0001**	C. Ob
Height (m)	1.70 (2–2)	1.70 (2–2)	1.64 (2–2)	1.69 (2–2)	0.240	
BMI (kg/m²)	23 (23–24)	32 (29–34)	40 (39–42)	32 (28–34)	**<0**.**0001**	C. Ob
BSA (m²)	1.80 (1.74–1.89)	2.05 (1.92–2.28)	2.30 (2.17–2.46)	2.10 (1.91–2.22)	**<0**.**0001**	C. Ob
Hip circumference (cm)	97 (95–100)	111 (106–117)	133 (128–138)	109 (100–116)	**<0**.**0001**	C. Ob
Waist circumference (cm)	82 (79–84)	105 (96–113)	116 (107–136)	103 (94–117)	**<0**.**0001**	C. Ob
**Biological parameters**						
HbA1c	5.5 (5.2–5.7)	5.9 (5.7–6.0)	5.9 (5.6–6.2)	7.7 (6.5–8.5)	**<0**.**0001**	C. MS. Ob
IL6	1.56 (1.03–2.01)	2.26 (1.32–3.54)	2.6 (2.03–3.18)	1.87 (1.50–3.23)	0.410	
Leptin	7.0 (2.9–16.6)	14.5 (7.5–32.9)	60.1 (43.2–70.4)	11.2 (7.8–28.3)	**<0**.**0001**	Ob
Adiponectin	5.6 (3.7–8.5)	3.7 (2.9–6.0)	6.3 (4.5–7.7)	3.1 (2.8–4.2)	**0**.**002**	Ob
CRP	1.9 (1.1–3.4)	1.6 (0.7–5.3)	5.8 (3.3–14.6)	2.1 (0.7–3.3)	**0**.**035**	NS
Total fat mass (kg)	14 (11–17)	31 (25–39)	49 (43–59)	30 (25–38)	**<0**.**0001**	C. Ob
Fat mass proportion (%)	21 (17–29)	37 (28–42)	48 (44–51)	37 (29–42)	**<0**.**0001**	C. Ob
Visceral fat mass (kg)	7 (6–9)	14 (11–17)	15 (13–17)	12 (10–15)	**<0**.**0001**	C. Ob
**CMR global LV parameters**						
LVEF (%)	63 (56–67)	68 (59–71)	61 (56–66)	61 (55–65)	**0**.**009**	MS
LV mass (g)	92 (79–114)	101 (86–123)	98 (83–117)	110 (88–133)	0.402	
LV mass index (g/m²)	33 (31–37)	32 (28–36)	26 (23–29)	29 (25–33)	**<0**.**0001**	C
LV EDV (mL)	172 (132–192)	145 (128–167)	156 (142–170)	149 (119–161)	0.102	
LV EDV index (mL/m²)	92 (75–108)	70 (63–76)	69 (64–73)	70 (56–77)	**<0**.**0001**	C
**CMR left atrial parameters**						
Left atrial volume (mL)	67 (48–73)	60 (49–75)	67 (51–73)	60 (48–72)	0.506	
Left atrial volume index (mL/m²)	35 (25–40)	28 (25–35)	25 (24–33)	27 (24–34)	**0**.**039**	C
Groove EAT volume (mL)	2.8 (2.3–3.1)	4.5 (3.5–6.0)	4.0 (3.3–4.8)	3.5 (3.0–4.4)	**<0**.**0001**	C. MS
Groove EAT volume index (mL/m²)	1.5 (1.3–1.7)	2.1 (1.6–2.9)	1.7 (1.5–1.9)	1.7 (1.4–1.9)	**0**.**001**	MS
LA GLS reservoir (%)	43 (36–44)	39 (33–44)	39 (36–46)	35 (31–39)	**0**.**001**	C. MS. Ob
LA GLS booster (%)	19 (18–21)	19 (17–21)	19 (16–21)	18 (15–20)	**0**.**032**	NS
Anterior GEAT native T1 (ms)	276 (256–305)	265 (247–282)	262 (237–276)	248 (233–266)	**0**.**002**	C

Median and 25th–75th percentile values are given for continuous variables and frequencies for dichotomous variables. Pairwise comparisons with Bonferroni correction for the diabetes group: the name of the group with significant (*P* < 0.05) pairwise difference with the diabetes group is indicated as follows: C, control, MS, metabolic syndrome, Ob, obesity. Pairwise comparisons are provided when the global ANOVA is significant (*P* < 0.05).

^*^(P) statistical significance for ANOVA across all groups except sex (*χ*^2^ test).

The clustering analysis of the whole population showed controls, diabetes, and MSD to be well differentiated with the obese patients being centrally distributed with varied degrees of overlap with the diabetic or MSD group (see *Figure 4A*). Random forest machine learning algorithm validated the prediction power as well, with an out-of-bag error of 0.19 as evident by the confusion matrix. Taken together, these results indicate that GEAT is a biomarker that may allow to discriminate the cardiometabolic profile between metabolic disorders.

### Evidence for atrial cardiomyopathy in patients with diabetes

**Figure 3:**
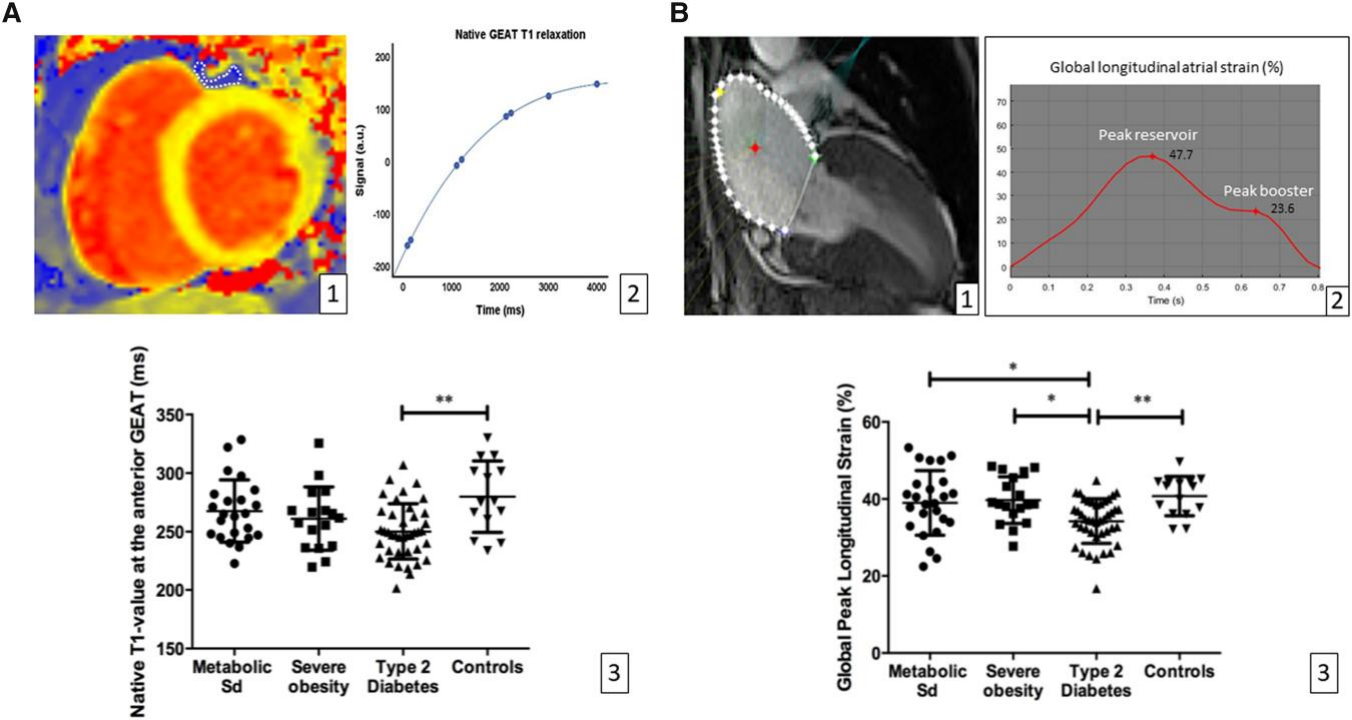
Native GEAT T1 relaxation time measurement (short-axis native MOLLI T1 map with blue surface overlay in the anterior interventricular groove T1; *A1*). T1 relaxation curve with T1-fitting curve; *A2*) and distributions of T1 relaxation times according to cardiometabolic group (*A3*). Measurement of LA peak GLS on a two-chamber SSFP view using feature tracking (CardioTrack LIB INSERM/Sorbonne Université; *B1*) and resulting strain curve with reservoir and booster function peaks (*B2*). LA peak longitudinal strain distributions according to cardiometabolic groups (*B3*).

In diabetic patients, in addition to increased volume, the composition of GEAT was altered as shown by the reduced value of native T1 relaxation compared with the control group (*Figure 3A*). Despite a similar trend, this difference did not reach statistical significance when comparing patients with diabetes to those with obesity or MSD. In addition, patients with obesity or MSD did not differ significantly from the control group. Furthermore, the peak GLS of the LA, an early marker of atrial dysfunction,^14^ was only reduced in T2D patients (*Figure 3B*). Despite a similar trend for the booster pump function, this did not reach statistical significance in pairwise comparisons. Note that the alteration in GLS remained statistically significant in the diabetes group even after adjustment for LV mass, LA end-diastolic volume, age, and sex, the main determinants of LA strain.

**Figure 4:**
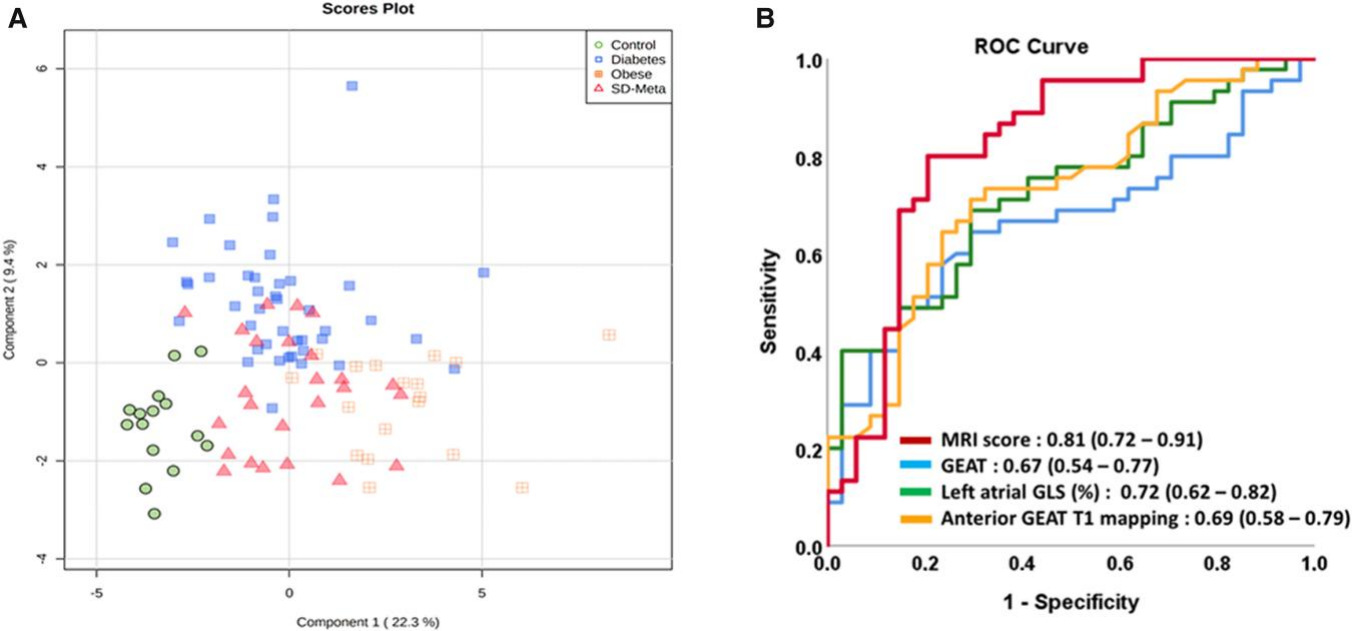
Clustering of the diabetic group from the overall study population using the diabetic CMR prediction score (*A*). ROC analysis comparing the ability of the diabetic CMR score to identify the diabetic patients based on atrial CMR parameters (*B*).

In order to test if T2D patients could be identified by distinct LA alterations, we developed a GEAT score by logistic regression combining GEAT volume, groove T1 relaxation time, and LA peak GLS that were significant associates of diabetes in the regression analysis. The PLS-DA was applied to discriminate T2D from the rest of the cohort, and we found that the GEAT diabetes score was the major contributor in the discrimination results as shown through the PCA loadings (*Figure 4A* and *B*). The area under the receiver operating curve (ROC) analysis of the GEAT diabetes score excellently predicted the T2D patients with a value of 0.81 (Se: 77%, Spe: 80%; 95% CI 0.72–0.91, *P* < 0.0001) from the rest of the cohort (*Figure 4B*). In parallel, we performed random forest machine learning algorithm with 3000 trees to validate the GEAT score’s predictive power. The CMR score was defined as CMR score = 11.886 + (−1.207 ∗ GEAT volume index) + (−0.112 ∗ LA PLS) + (−0.056 ∗ GEAT T1).

## Discussion

In this study, we report that (i) the pure EAT located in the AV groove can be easily and accurately measured from conventional cine CMR images in all patients and used as a surrogate for the atrial EAT; (ii) in a cohort of patients with distinct metabolic status, it is possible to discriminate between obese, diabetes, or MSD patients using GEAT in combination with other CMR parameters.

Echocardiography has been proposed to measure EAT thickness.^1^ However, this observer-dependent technique is impaired by the reliance on a 1D measurement prone to location variability while EAT is an anatomically complex 3D tissue with high regional heterogeneity. CT has the advantage of full isotropic and sub-millimetric anatomical coverage with good native sensitivity to adipose tissue densities useful to characterize, respectively, adipose tissue volume and content, which may benefit from recent semi-automated and artifical intelligence-based volumetric segmentation algorithms.^15^ However, adipose tissue density assessment remains challenging as it depends on several factors including contrast medium use (type, dose, and timing) and underlying tissue level parameters such as microvasculature, fibrosis, and inflammation processes, which impact contrast medium kinetics. Drawbacks of CT include the use of radiation and the lack of simultaneous high-resolution assessment of myocardial dynamics. CMR, despite lower spatial resolution, has the ability to non-invasively provide precise atrial volumes and quantitative assessment of deformation measured as atrial strain as well as sensitive tissue characterization using T1 relaxation mapping. Finally, all these imaging approaches cannot discriminate exactly between the different localizations and composition of paracardiac adipose tissue. Assessing GEAT has the dual benefit of being highly standardizable when using the established long-axis views and targeting specific, pure EAT with demonstrated physiopathological value.

We found that GEAT volume is increased in obese patients as already reported for EAT.^16^ This finding together with the high correlation between GEAT and atrial EAT volumes indicates that the former can be used as a proxy to the latter. Furthermore, there is evidence for enhanced clinical and biological relevance in targeting GEAT instead of atrial or total EAT as aforementioned. Patients with diabetes or MSD also show an increased GEAT volume despite a lower total body mass than obese subjects. Thus, various clinical conditions could be associated with the accumulation of EAT independently of abnormal ectopic fat deposition. Indeed, EAT abundance is increased in patients with insulin resistance and impaired glucose tolerance. In the same line, obese patients with T2D show a greater propensity for ectopic and visceral fat deposition.^17^ In obese and glucose-intolerant mice, the atrium becomes adipogenic as a result of abnormal storage of dietary fat by the atrial myocardium.^18^ Changes in atrial working conditions are another condition associated with EAT accumulation.^11,19^ Therefore, various remote or local factors can trigger the expansion of EAT.

In diabetic patients, GEAT is not only more abundant, but also its structure and composition are abnormal as indicated by the reduced native T1 relaxation in CMR. There is other evidence for specific biological properties of the EAT in diabetic patients,^20^ such as differences in gene expression, notably those encoding inflammatory cytokines.^21^ Native T1 relaxation is dependent on the fat/water fraction and the biochemical characteristics of fat components within the adipose tissue of interest. The T1 relaxation time of adipose tissue obtained with 1.5 T has been shown to decrease in severely obese individuals compared with lean controls.^22^ CMR and MR spectroscopy performed on murine adipose tissue showed the possibility to distinguish brown and white adipose tissue signatures with lower levels of T1 and unsaturated triglycerides found in brown adipose tissue vs. white adipose tissue.^23^ Beyond water content, spin-lattice relaxation has been shown to be influenced by different fatty-acid chain lengths and degrees of saturation.^24^ Decreased microvascular density observed in T2D^25^ may also partially explain the observed T1 times as the T1 of blood *in vivo* is high relative to fat. We hypothesize that diabetic patients may display a phenotypical profile in CMR associating increased EAT volume, altered EAT structure and/or function as shown by decreased T1 relaxation times and decreased atrial myocardial function demonstrated by strain. LA strain has been shown to be an integrator of structural and functional properties of the myocardium such as fibro-fatty infiltration of atrial myocardium or metabolic stress.^26,27^ During diabetes, myocardial fibrosis could contribute to impaired deformation of the LA. In addition, alteration of glucose utilization by the atrial myocardium can contribute also to altered functional properties of the atria as demonstrated experimentally.^18^ This is in line with the by decreased peak longitudinal strain function and LA deformation in T2D patients.^28^

We found that combining GEAT volume and T1 with LA strain to generate a diabetes score, it was possible to identify T2D patients from a population with varied cardiometabolic risk profiles. Since its first definition,^29^ it is now clear that the concept of atrial cardiomyopathy covers a diversity of diseases and clinical conditions, such as atrial cardiomyopathy associated with heart failure, ageing, and mitral valve diseases.

### Limitations

We could not technically assess the microvascular component of GEAT in CMR because of spatial and temporal resolution issues intrinsic to *in vivo* imaging. It has been shown that paracrine inflammatory signals from the vascular wall, notably in the presence of atheroma plaque, impact perivascular adipose tissue, a phenomenon referred to as fat attenuation.^7^ Thus, we cannot directly account for GEAT imaging characteristics as it is partially determined by subclinical microvascular disease. In the same line, limited spatial resolution and potential motion correction-related artefacts at tissue interfaces may affect GEAT T1 relaxation times measurements and might explain the limited discrimination of different T1 values across the different pathological conditions. Here, the proposed method to measure GEAT is an estimate and not an exact volume measurement of the GEAT, which would have required high-resolution 3D SSFP not yet available in routine imaging. Nevertheless, the strength of the proposed method is precisely to be based on routine long-axis imaging in the standard multi-modal planes performed in all routine cardiac MRI exams. Furthermore, these planes allow a precise visualization and delineation of the groove borders as they sample the groove perpendicularly with respect to the groove centreline providing triangular-shaped groove EAT. Further studies in larger population samples may also benefit from 3D automated volumetric assessment of EAT merged with T1 and strain maps. Whether changes in GEAT groove and LA deformation observed in diabetic patients indicate a distinct atrial cardiomyopathy or are part of a global diabetic cardiomyopathy remains to be mechanistically determined.

## Conclusions

We found that GEAT volume is a proxy biomarker of the properties of atrial EAT that can be measured in routine cardiac CMR. GEAT volume and T1 combined with atrial deformation are altered in patients with metabolic diseases and able to identify different patterns of alteration with a singular signature related to atrial dysfunction in patients with diabetes. Overall, our study demonstrates the potential value of using multi-modality parameters for the early diagnosis of atrial cardiomyopathy. Further studies evaluating the value of this approach to identify patients at risk of AF and stroke are warranted.

## Data Availability

The data underlying this article will be shared on reasonable request to the corresponding author.

## Lead author biography

Stéphane N. Hatem is an MD in Cardiovascular diseases and PhD in cardiac physiology. He is full professor of cardiovascular physiology (Sorbonne-Université and Paris Hospital, APHP). Since 2004, he is director of INSERM/Sorbonne-university research units (2019-now: UMRs1166). Since 2019; he is director of one of the 6 University-Hospital institutes (IHU) created in France: the IHU-ICAN. He is the coordinator of the H2020 MAESTRIA network and has been partners in two European (FP7 EUTRAF and H2020 CATCHE ME) networks. He has been the European coordinator of a Leducq TransAtlantic Network (2009-2014).
